# Effects of the Interactions Between Food Additive Titanium Dioxide and Matrices on Genotoxicity

**DOI:** 10.3390/ijms26020617

**Published:** 2025-01-13

**Authors:** Su-Min Jeong, Han-Na Nam, Soo-Jin Choi

**Affiliations:** Department of Food Science & Technology, Seoul Women’s University, Seoul 01797, Republic of Korea; zzzin_pang@swu.ac.kr (S.-M.J.); nha0318@swu.ac.kr (H.-N.N.)

**Keywords:** titanium dioxide, food additives, interactions, matrices, genotoxicity, oxidative stress

## Abstract

Titanium dioxide (TiO_2_), a white color food additive, is widely used in bakery products, candies, chewing gums, soups, and creamers. Concerns about its potential genotoxicity have recently emerged, particularly following the European Union’s ban on its usage as a food additive due to its genotoxicity potential. Conflicting in vitro and in vivo results regarding its genotoxicity highlight the need for further in-depth investigation. Moreover, food additives can interact with food components or biological matrices, potentially altering their biological responses and genotoxicity. In this study, we evaluated the interactions between two different sizes of additive TiO_2_ particles and food or biological matrices, including albumin, fetal bovine serum (FBS), and glucose. The results showed that the hydrodynamic diameters of TiO_2_ increased upon interaction with albumin or FBS, but not with glucose. The presence of albumin or FBS reduced TiO_2_-induced cytotoxicity, oxidative stress, in vitro intestinal transport, and ex vivo intestinal absorption to untreated control levels, regardless of particle size. While TiO_2_ caused DNA damage in intestinal Caco-2 cells, the interactions with albumin or FBS significantly reduced the DNA damage to levels comparable to untreated controls. The DNA damage was closely related to oxidative stress caused by TiO_2_. These findings suggest that the interaction of TiO_2_ with albumin or FBS, resulting in increased hydrodynamic diameters, mitigates its cytotoxicity, oxidative stress, intestinal transport, and genotoxicity. Further investigation is required to fully understand the potential genotoxicity of TiO_2_ in food contexts.

## 1. Introduction

Titanium dioxide (TiO_2_), also known as titania, is an inorganic compound formed naturally through the interaction of titanium with oxygen. It is widely used as a white pigment in various products, including foods, paints, coatings, pharmaceutics, medicines, cosmetics, and toothpaste. In the food industry, TiO_2_ serves as a white color additive in items, such as bakery products, candies, chocolates, chewing gums, soups, and creamers. It is authorized as the food additive E171 in the European Union (EU) under Annex II of Regulation (EC) No. 1333/2008. However, the European Food Safety Authority (EFSA) reassessed the safety of TiO_2_ in 2021 and determined that its potential genotoxicity could not be ruled out based on new data, although the evidence for genotoxic effect was not conclusive. Consequently, TiO_2_ was considered no longer safe as a food additive [1]. Following EFSA’s reassessment, the European Commission banned the use of TiO_2_ as a food additive in 2022 [2]. On the other hand, the United States Food and Drug Administration (FDA) approved the use of TiO_2_ in food in 1966, under the specifications and conditions outlined in (21 CRF 73.575), including a restriction that its concentration must not exceed 1% by weight of the food [3]. In 2023, the FDA identified no concerns regarding potential genotoxicity of TiO_2_ based on available data and stated that studies by National Toxicology Program did not indicate cancer risk of TiO_2_ [4]. The Joint Food and Agriculture Organization of the United Nations (FAO)/World Health Organization (WHO) Expert Committee on Food Additives (JECFA) did not establish a limit on its intake, citing its insolubility, inertness, and low oral absorption as evidence of its safety [5]. The FAO/WHO JECFA reaffirmed a “not specified” acceptable daily intake (ADI) for TiO_2_ in November 2023 [6]. Other regulatory authorities, including the United Kingdom’s Food Standards Authority, Health Canada, and Food Standards Australia New Zealand have not aligned with EFSA’s assessment and continue to permit the use of TiO_2_ in food products due to insufficient in vivo and human data on its genotoxicity [7,8,9].

As mentioned above, there are conflicting opinions regarding the genotoxicity of the food additive TiO_2_. Concerns have been raised about the potential toxicity of food-grade TiO_2_, particularly its effect on intestinal inflammation or function, intestinal diseases or cancer, and embryonic development in mice [10,11,12,13]. A meta-analysis of in vivo and in vitro studies concluded that TiO_2_ nanoparticles (NPs) induced genotoxic effects, including DNA damage, chromosomal aberrations, and gene mutations [14]. On the other hand, developmental oral toxicity studies involving six forms of TiO_2_ determined a no observed adverse effect level (NOAEL) of 1000 mg/kg [15]. A 90 d repeated oral toxicity study of E171 TiO_2_ also reported a NOAEL ranging from 100 to 1000 mg/kg, based on gene profile analyses [16]. Furthermore, recent standardized in vivo tests demonstrated that food additive TiO_2_ shows no genotoxic potential at doses up to 1000 mg/kg [17]. The oral absorption and bioavailability of food-grade TiO_2_ were found to be less than 0.1%, although oral absorption increased to 0.8% when interacting with food matrices [18]. These conflicting findings seem to be influenced by variations in test materials (food- or general grade, particle size, and surface characteristics), biological systems used (in vitro, in vivo, and matrices), and doses (concentrations).

TiO_2_ is well known for its photocatalytic activity, producing electrons that react with environmental molecules to generate free radicals, including reactive oxygen species (ROS) [19,20,21]. ROS, being highly reactive, can interact with cellular components, causing damage to DNA, RNA, proteins, and membranes, which may ultimately lead to cell death. Several studies have demonstrated that TiO_2_ exposure induces oxidative stress in cultured cells [22,23,24]. Concerns about the potential genotoxicity of TiO_2_ are strongly linked to its role in generating ROS and causing oxidative stress. Indeed, oral intake of TiO_2_ NPs was reported to increase ROS levels in the colon, triggering oxidative stress in mice [25]. Additionally, food-grade TiO_2_ may interact with other molecules in foods and biological fluids, forming a particle-molecule corona that alters its physicochemical property, biological responses, and toxicity [18,26,27]. The matrix covers the surface of the particles, leading to changes in particle size or modifications in surface charges. Such interactions between particles and food components or biological matrices can significantly influence its biological effects. For instance, oral absorption of food additive TiO_2_ has been shown to increase in the presence of albumin or glucose associated with reduced hydrodynamic diameters of TiO_2_ [18].

We hypothesized that the potential genotoxicity of TiO_2_, associated with its ROS-generation properties, could be mitigated through interactions with food or biological matrices. This study aimed to investigate the genotoxic potential of food additive TiO_2_ in various in vitro biological systems, including cultured cell lines and 2D/3D intestinal barrier models. Furthermore, we evaluated the impact of TiO_2_-matrix interactions on its genotoxicity.

## 2. Results and Discussion

### 2.1. Characterization of Food Additive TiO_2_ Interacted with Matices

**Figure 1:**
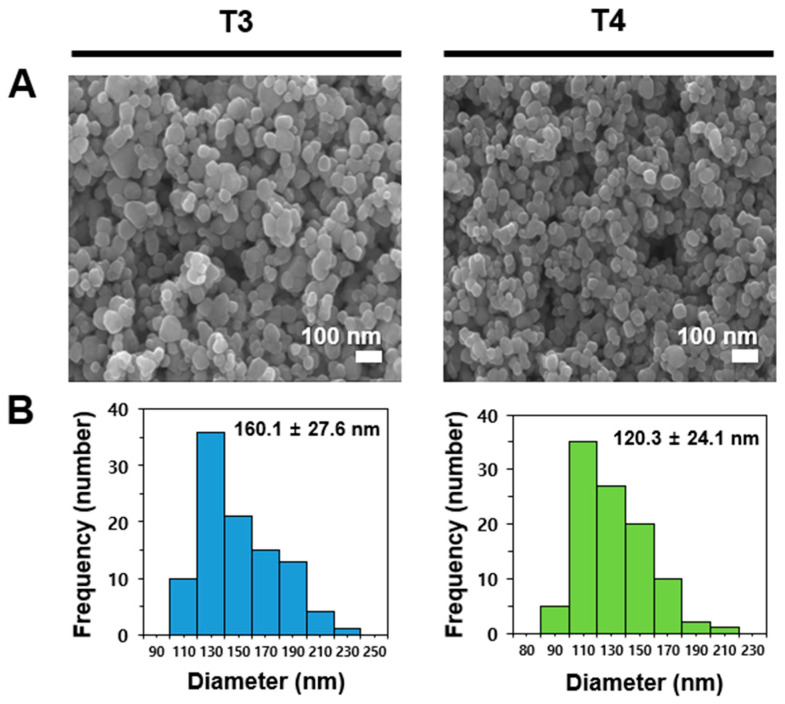
(**A**) Scanning electron microscopy (SEM) images and (**B**) size distribution of two differently sized TiO_2_ particles (T3 and T4). Particle size distribution was determined by randomly selecting 100 particles from SEM images.

For this study, we selected two differently sized food additive TiO_2_ particles, representing the largest (T3) and smallest (T4) particles among the five most widely commercially available types identified in our previous report [24]. The shape and constituent particle size of TiO_2_ particles were determined by scanning electron microscopy (SEM), showing average constituent particle sizes of 160.1 ± 27.6 nm and 120.3 ± 24.1 nm for T3 and T4, respectively, with irregular shape (Figure 1). The crystal structure was determined to be anatase forms for both particles [24].

The hydrodynamic diameters of two particles were measured in distilled and deionized water (DDW) and minimum essential medium (MEM) (Table 1), which reflect environments for characterization and cell culture experiments, respectively. The hydrodynamic diameters of T3 and T4 in DDW increased compared to its constituent particle size measured by SEM (Table S1), showing 304.7 ± 0.9 nm and 295.7 ± 2.9 nm for the former and latter, respectively, indicating aggregate formation under aqueous solution. The hydrodynamic radii in MEM were 440.9 ± 4.3 nm and 377.3 ± 2.8 nm for T3 and T4, respectively, and significantly increased compared to those in DDW. This result may be attributed to the interaction between particles and diverse biological matrices present in MEM. Meanwhile, the presence of food or biological matrices affected differently the hydrodynamic diameters of TiO_2_. Albumin and glucose were selected as food matrices due to their ability to increase oral absorption of TiO_2_ particles [18], and fetal bovine serum (FBS) was also included as a representative biological matrix. The interaction between TiO_2_ and FBS or albumin significantly increased the hydrodynamic diameters compared to those in DDW or MEM, whereas the hydrodynamic radii of TiO_2_ interacted with glucose was statistically same to those in DDW or MEM. It is likely that large molecules, such as FBS and albumin, increase the hydrodynamic diameters of TiO_2_ by forming large FBS– or albumin–particle corona [28,29]. The relatively decreased hydrodynamic diameters observed in the presence of glucose are likely to be related to the dispersant capacity of glucose for particles [30,31]. Various components in MEM, such as vitamins, minerals, amino acids, glucose, and others, can also help reduce particle aggregation to some extent. These results clearly suggest that the TiO_2_ interactions with food or biological matrices differently influence the hydrodynamic diameters and aggregate formation of TiO_2_ particles.

### 2.2. In Vitro Solubility in Digestive Fluids of Food Additive TiO_2_ Interacted with Matices

The interaction effects of TiO_2_ particles interacted with matrices on solubility were investigated in simulated digestive systems (saliva + gastric + intestinal fluids). The solubilities of T3 and T4 in distilled water (DW) were 0.15% and 0.18%, respectively, without significant differences (*p* > 0.05). The solubility levels of TiO_2_ particles interacted with FBS, albumin, or glucose were not affected, ranging from 0.11% and 0.18% without any statistical differences (*p* > 0.05). The results suggest that the oral solubility of TiO_2_ particles was extremely low, regardless of interacting matrix types or particle size. The low and slow dissolution properties of TiO_2_ particles in diverse biological fluids were demonstrated by several previous reports [18,32,33]. Indeed, TiO_2_ particles are generally considered to be insoluble and resistant to dissolution [34,35]. Hence, it seems that the solubility is not a crucial factor affecting the toxicity of TiO_2_ particles; rather, its biopersistence in particle forms may cause long-term health effects.

### 2.3. Cytotoxicity of Food Additive TiO_2_ Interacted with Matices

**Figure 2:**
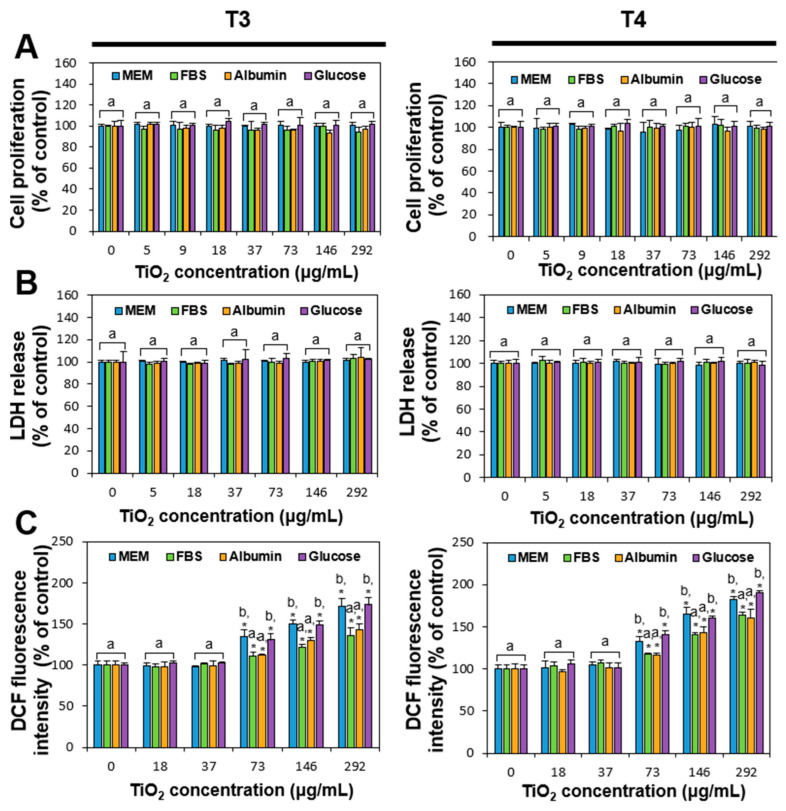
(**A**) Cell proliferation inhibition, (**B**) lactate dehydrogenase (LDH) release, and (**C**) reactive oxygen species (ROS) production caused in Caco-2 cells exposed to TiO_2_ particles (T3 and T4) interacted with food or biological matrices. Different lowercase letters (a, b) above bars denote significant differences among different matrices interacted (untreated control, MEM, FBS, albumin, and glucose) (*p* < 0.05). * denotes significant difference compared to untreated control cells (*p* < 0.05). Abbreviation: DCF, dichlorofluorescein fluorescence.

The effects of the TiO_2_ interactions with food or biological matrices on cell proliferation, lactate dehydrogenase (LDH) release, and ROS generation were evaluated in human intestinal Caco-2 cells. The maximum concentration of 292 μg/mL was chosen based on maximum usage from Manufacturing Report (2018–2019) and daily intake levels from Item and National Food and Nutrition Statistics (2017) in the Republic of Korea, and adjusted for cell experiment conditions reflecting volume of human intestinal fluids [36,37]. The results demonstrate that both TiO_2_ particles with or without food or biological matrices did not affect cell proliferation and LDH release level at the highest concentration tested (Figure 2), which is consistent with our previous report [24]. Meanwhile, TiO_2_ particles significantly increased ROS generation in a concentration-dependent manner at above 73 μg/mL, regardless of particle size (T3 or T4) (Figure 2C). It is interesting to note that ROS generation significantly increased when TiO_2_ particles dispersed in MEM or glucose compared to those in FBS or albumin. This result can be explained by the hydrodynamic radii of TiO_2_ interacted with matrices (Table 1), showing larger hydrodynamic diameters in FBS or albumin than those in MEM or glucose. Hence, TiO_2_ particles in MEM or glucose had small hydrodynamic diameters, which may lead to high cytotoxicity. It was demonstrated that small-sized particles induced higher cytotoxicity than large particles [38,39,40]. In other words, biological matrices, such as FBS and albumin, can reduce ROS generation by TiO_2_ particles through interactions that form large matrix–particle aggregates.

### 2.4. Antioxidant Enzyme Activity of Food Additive TiO_2_ Interacted with Matices

**Figure 3:**
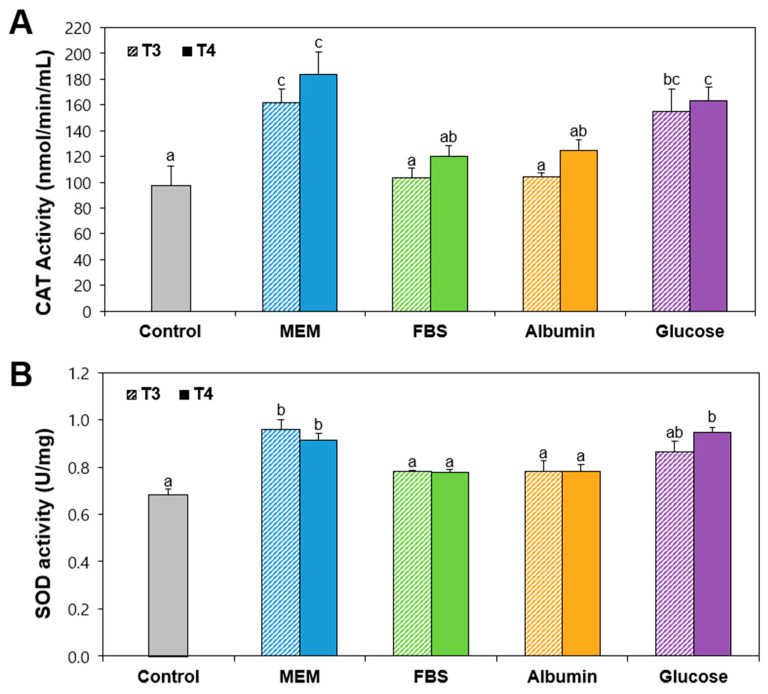
(**A**) Catalase (CAT) and (**B**) superoxide dismutase (SOD) activities in Caco-2 cells exposed to TiO_2_ particles (T3 and T4) interacted with food or biological matrices. Control represents the basal antioxidant enzyme activities in Caco-2 cells without particles. Different lowercase letters (a, b, c) above bars denote significant differences among different matrices interacted (untreated control, MEM, FBS, albumin, and glucose) (*p* < 0.05).

The activities of catalase (CAT) and superoxide dismutase (SOD) in Caco-2 cells exposed to TiO_2_ particles were investigated because both T3 and T4 particles induced ROS in a concentration-dependent manner (Figure 2C). Figure 3 demonstrates that the activities of both CAT and SOD significantly increased by TiO_2_ particles prepared in MEM or glucose, whereas the enzyme activities by TiO_2_ interaction with FBS or albumin were statistically same to untreated control cells, regardless of particle size (T3 and T4). This result suggests the induction of oxidative stress by TiO_2_ particles, especially when interacting with MEM or glucose. In other words, TiO_2_ in the presence of FBS or albumin can reduce oxidative stress.

### 2.5. In Vitro Intestinal Transport of Food Additive TiO_2_ Interacted with Matices

**Figure 4:**
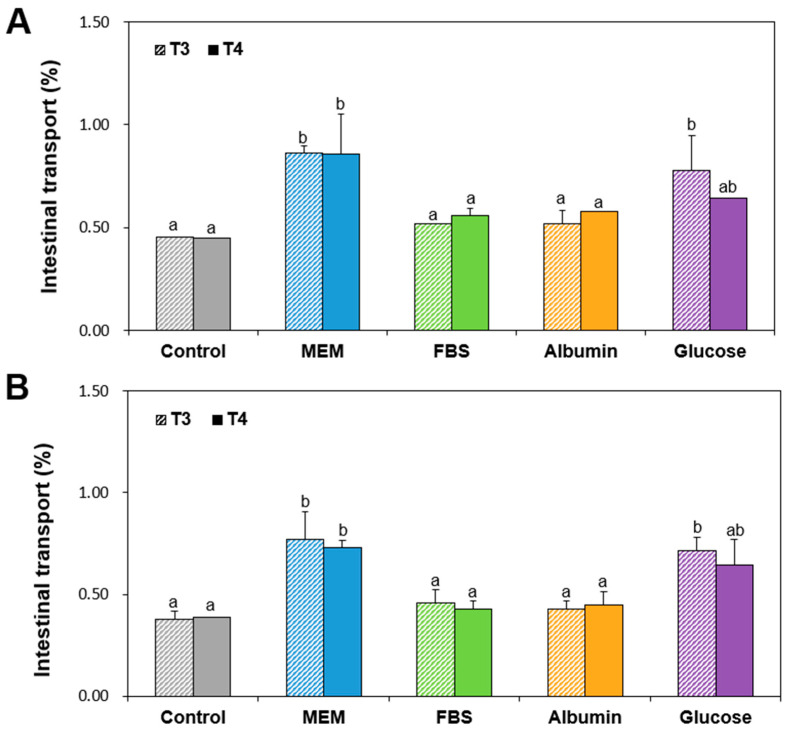
In vitro intestinal transports of TiO_2_ particles (T3 and T4) interacted with food or biological matrices through (**A**) Caco-2 monolayers and (**B**) follicle-associated epithelial (FAE) models. Control represents the basal Ti levels in the two models without particles. Different lowercase letters (a, b) above bars denote significant differences among different matrices interacted (untreated control, MEM, FBS, albumin, and glucose) (*p* < 0.05).

The TiO_2_ interaction effects on intestinal transport were evaluated using a Caco-2 monolayer model reflecting intestinal tight junction barrier and follicle-associated epithelial (FAE) models, representing microfold (M) cells found in the intestinal epithelium which play a role in immune response. The transport amounts of both T3 and T4 particles dispersed in MEM or glucose were significantly higher than those in FBS or albumin in both Caco-2 monolayers and FAE models without statistical significances between T3 and T4 (Figure 4). It is worth noting that transported levels of TiO_2_ though Caco-2 monolayers and M cells in the presence of FBS or albumin were extremely low, below 0.13%. These results seem to be strongly related to the hydrodynamic radii of TiO_2_ particles, showing increased hydrodynamic radii in FBS or albumin, but relatively small diameters in MEM or glucose (Table 1). Indeed, increased transport levels of small particles compared to bulk particles were demonstrated in several studies [41,42,43]. No remarkable differences in transport amounts between T3 and T4 particles were found, which is consistent with our previous research [18]. Meanwhile, total transport levels of TiO_2_ particles through the two intestinal barrier models were less than 1% in all cases, indicating extremely low in vitro intestinal transport at real intake levels. These results also imply that the interactions between TiO_2_ and FBS or albumin can decrease in vitro intestinal transport levels.

### 2.6. Ex Vivo Intestinal Absorption of Food Additive TiO_2_ Interacted with Matices

**Figure 5:**
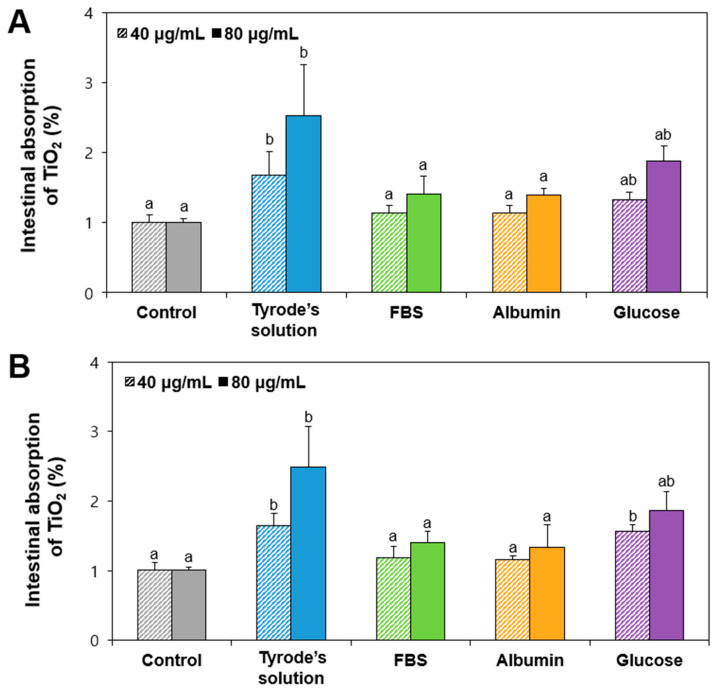
Ex vivo intestinal absorption of TiO_2_ particles ((**A**) T3; (**B**) T4) interacted with food or biological matrices at two different doses using an everted gut sac model. Control represents basal Ti levels in everted gut sac without particles. Different lowercase letters (a, b) above bars denote significant differences among different matrices interacted (untreated control, MEM, FBS, albumin, and glucose) (*p* < 0.05).

Ex vivo intestinal absorption amounts of TiO_2_ particles were evaluated using an everted gut sac model as previously reported [44,45,46]. The dose of 40 μg/mL was chosen based on actual oral intake levels in rats (converted from human intake) and ex vivo experimental everted rat gut sac conditions (gut sac length and test volume). A higher dose of 80 μg/mL was also included to investigate potential effect of high exposure on intestinal absorption. The results show similar tendency to in vitro intestinal transport results (Figure 4). Higher intestinal absorptions of TiO_2_ particles in Tyrode’s solution (without matrices) or glucose were found relative to those in FBS or albumin (Figure 5). These results can be also explained by increased hydrodynamic diameters of TiO_2_ in FBS or albumin relative to those in glucose (Table 1). No effect of particle size (T3 and T4) or dose was found (*p* > 0.05). Notably, the maximum intestinal absorption levels of TiO_2_ were below 1.5% and decreased to basal levels (comparable to those without particles) when interacting with FBS or albumin. These findings imply that the interactions between TiO_2_ particles and food or biological matrices, such as albumin or FBS, can decrease in vitro and ex vivo intestinal absorption, potentially contributing to their low oral toxicity.

### 2.7. DNA Damage Caused by Food Additive TiO_2_ Interacted with Matices

**Figure 6:**
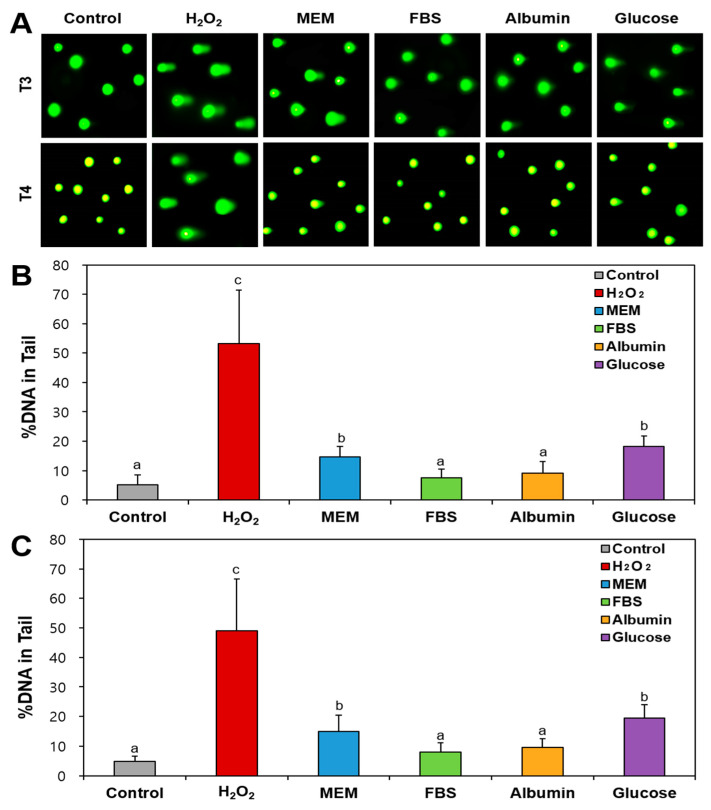
DNA damage caused by TiO_2_ particles (T3 and T4) interacted with food or biological matrices assessed with comet assay in Caco-2 cells. (**A**) Representative images of Caco-2 cells treated with TiO_2_. Images were magnified at 20×. Percentage DNA values in tails exposed to (**B**) T3 and (**C**) T4. Control represents DNA (%) in tails of untreated cells without particles. Different lowercase letters (a, b, c) above bars denote significant differences among different matrices interacted (untreated control, MEM, FBS, albumin, and glucose) (*p* < 0.05).

The potential genotoxicity was assessed with the comet assay, a sensitive and cost-effective technique used to measure DNA damage. The percentage (%) of total DNA in the comet tail serves as a quantitative indicator of the frequency of DNA breaks or fragments in cells. Figure 6 shows that both T3 and T4 particles in MEM caused DNA damage in Caco-2 cells, even if the damage levels were significantly much lower than well-known positive control for DNA damage, H_2_O_2_. The result indicates that food additive TiO_2_ particles have potential to cause DNA damage, probably related to their ROS generation and oxidative stress (Figure 2C and Figure 3). Several reports demonstrated DNA damage caused by TiO_2_ particles in cultured cell lines [47,48,49]. On the other hand, the statistically same DNA damage level by TiO_2_ particles was found when they interacted with glucose. Interestingly, total DNA (%) in comet tail significantly decreased by TiO_2_ particles dispersed in FBS or albumin, regardless of particle size. These results can be explained by decreased in vitro intestinal absorption of TiO_2_ particles (Figure 4) related to their large hydrodynamic diameters in FBS or albumin (Table 1). These findings suggest that the interactions between TiO_2_ particles and food or biological matrices may reduce the genotoxicity potential of TiO_2_.

### 2.8. 8-Hydroxy-2′-Deoxyguanosine (8-OHdg) Production by Food Additive TiO_2_ Interacted with Matices

**Figure 7:**
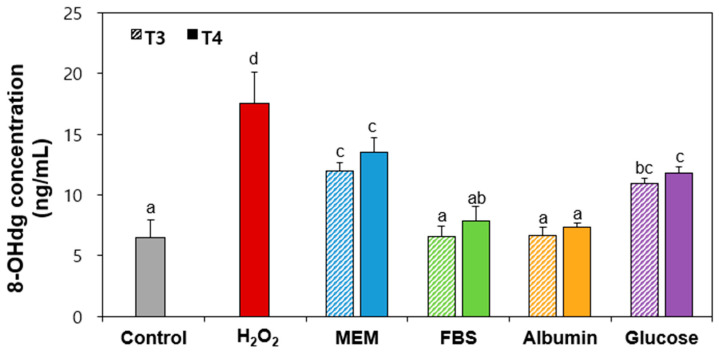
8-hydroxyl-2′-deoxyguanosine (8-OHdg) generated by TiO_2_ particles (T3 and T4) interacted with food or biological matrices in Caco-2 cells. Control represent basal 8-OHdg levels of untreated cells without particles. Different lowercase letters (a, b, c, d) above bars denote significant differences among different matrices interacted (untreated control, MEM, FBS, albumin, and glucose) (*p* < 0.05).

8-OHdg is one of the most representative derivatives of DNA formed by ROS or photodynamic action, and is used as a biomarker of oxidative stress-induced DNA damage and cancers [50,51]. Figure 7 shows that both TiO_2_ particles in MEM or glucose significantly produced 8-OHdg compared to untreated control cells, whereas TiO_2_ interacted with FBS or albumin did not increase 8-OHdg levels. No effect of particle size was found (*p* > 0.05). This result clearly suggests that TiO_2_ particles generate ROS, which can lead to DNA damage associated with oxidative stress.

## 3. Materials and Methods

### 3.1. Materials

Two different food additive E171 TiO_2_ were provided from commercial manufacturers, which were chosen as representative largest and smallest particle based on our previous report [24]. TiO_2_ dispersions (1 mg/mL) in deionized and distilled water (DDW) were stirred for 30 min and bath-sonicated for 5 min (160 Watts, Bransonic 580, Branson Ultrasonics, Danbury, CT, USA) prior to experiments. FBS was purchased from Welgene Inc. (Gyeongsan, Gyeongsangbuk-do, Republic of Korea). Albumin and glucose were obtained from Sigma-Aldrich Co., Ltd. (St. Louis, MO, USA). For interaction study, the stock solutions (292 μg/mL) were prepared in 1% FBS, 1% albumin, and 1% glucose, respectively, and dispersed as describe above. The three matrices were prepared in DDW and minimum essential medium (MEM), respectively, for comparison.

### 3.2. Characterization

Constituent particle size and shape were determined by field emission-scanning electron microscopy (SEM, JSM-7100F, JOEL, Tokyo, Japan) as previously reported [52]. Hydrodynamic radii of TiO_2_ particles were measured with a Zetasizer Nano System (Malvern Instruments, Worcestershire, UK) via light scattering (DLS). X-ray diffraction (XRD) pattern analysis was applied to determine the crystalline phase using Ni-filtered CuKα radiation (D2phaser, Bruker AXS Inc., Madison, WI, USA) [24].

### 3.3. In Vitro Solubility in Digestive Fluids

The solubility of TiO_2_ particles in simulated gastrointestinal digestive fluids consisting of simulated salivary fluid (SSF), simulated gastric fluid (SGF), and simulated intestinal fluid (SIF) was investigated according to INFOGEST 2.0 protocol [53]. Briefly, 1 mL of TiO_2_ particles (2 mg/mL) suspended in the absence or presence of matrices were incubated in 1 mL of SSF at 37 °C for 2 h, followed by incubation in 1 mL of SGF and 1 mL of SIF at 37 °C for 2 h, respectively. After centrifugation (16,000× *g*) for 15 min, Ti levels in supernatants were analyzed by inductively coupled plasma-atomic emission spectroscopy (ICP-AES) as described in Section 3.9. ICP-AES Analysis.

### 3.4. Cell Culture

Human intestinal epithelial Caco-2 cells and non-adherent human Burkitt lymphoma Raji B cells were purchased from Korean Cell Line Bank (Seoul, Republic of Korea). Caco-2 cells and Raji B cells were maintained in MEM and RPMI 1640 medium, respectively, supplemented with heat-inactivated FBS (10%), penicillin (100 units/mL), and streptomycin (100 μg/mL) under 5% CO_2_ atmosphere at 37 °C.

### 3.5. Cytotoxicity

The effects of TiO_2_ on cell proliferation and membrane damage were investigated with water-soluble tetrazolium salt-1 (WST-1; Roche, Molecular Biochemicals, Manheim, Germany) and a CytoTox 96 Non-Radioactive Cytotoxicity assay (Promega, Madison, WI, USA), respectively. For WST-1 assay, Caco-2 cells (1 × 10^4^ cells/100 μL) were treated with TiO_2_ with or without matrices for 24 h. The cells were further incubated for 4 h after addition of WST-1 solution (10 μL), and the absorbance at 440 nm was measured using a microplate reader (Infinite^®^ M Plex, Tecan, Männedorf, Switzerland).

Cell membrane damage was evaluated by incubating Caco-2 cells (4 × 10^4^ cells/mL) with TiO_2_ in the absence or presence of matrices for 24 h. The cell culture medium (50 μL) was used for reaction with substrate solution (50 μL) for 30 min at room temperature. The absorbance at 492 nm was measured using a microplate reader (Infinite^®^ M Plex, Tecan) after addition of stop solution (50 μL).

The ROS generation inside cells was monitored with a peroxide-sensitive fluorescent probe, 20,70-dichlorofluorescein diacetate (H_2_DCFDA; Molecular Probes Inc., Eugene, OR, USA). Caco-2 cells (1 × 10^4^ cells/100 μL) were exposed to TiO_2_ in the absence or presence of matrices. After 24 h, the cells were further incubated with H_2_DCFDA for 30 min at 37 °C in the dark. Dichlorofluorescein fluorescence (DCF) inside cells was monitored using a microplate reader (Infinite^®^ M Plex, Tecan) after washing with phosphate buffered saline (PBS) at 485 nm and 530 nm for excitation and emission wavelengths, respectively. All experiments were conducted in triplicate on two different days.

### 3.6. Antioxidatn Enzyme Activity

Cells (1 × 10^6^ cells/2 mL) were exposed to TiO_2_ (292 μg/mL) in the absence or presence of matrices for 24 h. The antioxidant enzyme activities of CAT and SOD were evaluated [54,55], using a CAT assay kit (Cayman Chemical Co., Ann Arbor, MI, USA) and chemical SOD assay kit (Cayman Chemical Co., Ann Arbor, MI, USA), respectively, according to the manufacturer’s protocols. The experiments were conducted in triplicate on two different days.

### 3.7. In Vitro Intestinal Transport

A FAE model, mimicking M cells, was prepared as previously described [52,56]. Caco-2 cells (1 × 10^6^ cells/well) were cultured on apical insert slides. After 14 days, Raji B cells (1 × 10^6^ cells/well) cultured in Dulbecco’s modified eagle’s medium (DMEM) were placed in basolateral insert parts, and further cultured for 5 days. The coculture was stopped when the transepithelial electrical resistance (TEER) values were ranged from 150 Ω cm^2^ to 200 Ω cm^2^. The medium containing TiO_2_ (292 μg/mL) with or without matrices was placed on apical inserts and incubation for 24 h was carried out. The TiO_2_ transported into basolateral solutions was quantified using ICP-AES.

A Caco-2 monoculture model, representing the intestinal epithelial tight junction barrier was prepared as previously described [52,56]. Caco-2 cells (4.5 × 10^5^ cells/well) were cultured on apical inserts for 21 days (TEER values ≥ 300 Ω cm^2^), and the medium containing TiO_2_ (292 μg/mL) with or without matrices was placed on apical inserts for 24 h. The TiO_2_ transported into basolateral medium was quantified using ICP-AES. The experiments were conducted in triplicate on two different days.

### 3.8. Ex Vivo Intestinal Absorption

Male Sprague Dawley rats (nine weeks old) were provided by Koatech Co. (Pyeongtaek, Gyeonggi-do, Korea). The rats were housed in laboratory cages within a ventilated clean rack maintained at 20 ± 2 °C and 60 ± 10% relative humidity, under a 12 h light–dark cycle. The rats were provided with unrestricted access to a standard laboratory diet and water. The animals were acclimatized for 7 d prior to the experiments. All animal experiments were performed in accordance with guidelines of the Institutional Animal Care and Use Committee (IACUC) of Seoul Women’s University. The protocols used in this study were approved by the IACUC of Seoul Women’s University (SWU IACUC 2024A-1).

Ex vivo everted small intestinal sacs were prepared as previously described by Gu et al. [44,45,46]. In brief, four male rats were euthanized using CO_2_ following overnight fast with access to water. The small intestines were obtained and washed three times with Tyrode’s solution (0.8 g NaCl, 0.02 g KCl, 0.02 g CaCl_2_, 0.01 g MgCl_2_, 0.1 g NaHCO_3_, 0.005 g NaH_2_PO_4_, and 0.1 g glucose in 100 mL DW). The small intestines were then cut into 5 cm sections in length and everted using a puncture needle with a diameter of 0.8 mm. After clamping one end, the everted intestinal sacs were filled with 200 μL of Tyrode’s solution and secured with silk braided sutures. The sacs were then incubated with 3 mL of the samples, with or without matrices, in a 6-well plate under a humidified 5% CO_2_ atmosphere at 37 °C for 2 h. The solutions inside the sacs were collected, and the amounts of absorbed TiO_2_ particles were analyzed using ICP-AES as described in Section 3.9. ICP-AES Analysis. The experiments were conducted with five repetitions.

### 3.9. ICP-AES Analysis

TiO_2_ amounts was quantified by measuring total Ti levels using ICP AES (Perkin-Elmer, Avio 550, Shelton, CT, USA) as previously reported [57]. Biological samples (0.2 g) were digested in HF-resistant perfluoroalkoxy microwave digestion vessels using a microwave system (ETHOS EASY, Milestone Srl, Sorisole, Italy). The digestion process utilized 70% HNO_3_ (6 mL) and 40% HF (2 mL). The microwave system was set at 1800 W and programmed to irradiate for 15, 10, and 30 min to reach 120 °C, 160 °C, and 210 °C, respectively. Then, holding at 210 °C for 1 min and cooling at 25 °C followed. Finally, the samples were diluted to appropriate volumes with DDW for ICP-AES analysis: 334.94 nm wavelength, 1500 W radiofrequency power, and 12 L/min plasma gas flow.

### 3.10. Comet Assay

Cells (1 × 10^6^ cells/2 mL) were incubated with TiO_2_ (292 μg/mL) in the absence or presence of matrices for 24 h. H_2_O_2_ (100 μM) was used as a positive control. The cells were washed with PBS, detached with scraper after treatment with 1 mL of 5 mM ethylene diamine tetraacetic acid (in PBS) for 40 s, centrifuged, and re-suspended (1 × 10^5^ cells/1 mL) in ice-cold PBS. A comet assay was then carried out with a kit purchased from R&D systems (Minneapolis, MN, USA) according to the manufacturer’s protocol. The cell suspensions were mixed with low melting point agarose at a ratio of 1:10 (*v*/*v*), and 75 μL of the solution was immediately loaded onto comet slide. The slides were placed in a dark room at 4 °C for 30 min for gelation and immersed in a cold lysis solution 4 °C. After 1 h, the lysis solution was removed, and the slides were immersed in alkaline solution (pH > 13) for 30 min in the dark at room temperature. After removing the alkaline solution, the slides were electrophoresed at 20 V and 300 mÅ at 4 °C for 40 min in the dark. The slides were then washed with DDW, immersed in 70% ethanol for 5 min, air-dried, and stained with 100 μL of Cygreen dye for 30 min in the dark at 4 °C. Finally, the slides were analyzed using Axioskop 2 plus fluorescent microscope and Cometscore 2.0 imaging software (Carl Zeiss, Oberkochen, Germany). The comet tail lengths were digitally analyzed and percent (%) DNA in the tail (the fraction of DNA in the tail divided by the total amount of DNA) was calculated. The experiments were conducted in triplicate on two different days.

### 3.11. 8-OHdG Assay

Cells (1 × 10^6^ cells/2 mL) were exposed to TiO_2_ (292 μg/mL) in the absence or presence of matrices for 24 h. H_2_O_2_ (100 μM) was used as a positive control. The DNA was then extracted using the AccuPrep^®^ Genomic DNA Extraction kit (Bioneer, Daejeon, Republic of Korea) following the manufacturer’s protocols. The extracted DNA (20 μg) was denatured at 95 °C for 5 min and cooled on ice. Nuclease P1 (0.1 μL) was added to the denatured DNA and incubated for 1 h at 50 °C for nucleoside formation by adjusting pH at 5.3 with 20 mM sodium acetate. The mixture was incubated with alkaline phosphatase at 37 °C for 1 h for phosphate decomposition. The pH of the mixture was then adjusted to range from 7.5 to 8.5 and boiled for 10 min. The prepared samples were stored on ice before analysis. Oxidative stress biomarkers 8-OHdG, an RNA nucleoside which is an oxidative derivative of guanosine, was quantified using 8-OHdG enzyme-linked immunosorbent assay (ELISA) kit (Abcam, Cambridge, UK) according to the manufacturer’s protocol. The experiments were conducted in triplicate on two different days.

### 3.12. Statistical Analysis

All results were presented as mean ± standard deviation. One-way analysis of variance (ANOVA) with Tukey’s test was carried out using SAS Ver. 9.4 (SAS Institute, Cary, NC, USA) to assess the significant differences between group means tested. Statistical significance was defined as a *p*-value of <0.05. Normality of distribution and homogeneity of variation were tested using Shapiro–Wilk and Levene’s tests, respectively, prior to ANOVA analysis.

## 4. Conclusions

Interactions between two differently sized food additive TiO_2_ and food (albumin and glucose) or biological (FBS) matrices were investigated in terms of hydrodynamic diameters, cytotoxicity, intestinal transport, and genotoxicity. The results show that TiO_2_ interacted with FBS or albumin, leading to increased hydrodynamic radii, which in turn reduced cytotoxicity, intestinal transport, and DNA damage. The genotoxicity effects of TiO_2_ were closely associated with oxidative stress. These findings suggest that the interactions between TiO_2_ and food or biological components can affect biological responses and genotoxicity, potentially contributing to its safety as a food additive. Further studies are required to fully assess the potential genotoxicity of TiO_2_ in food products.

## Figures and Tables

**Table 1:** Hydrodynamic diameters of TiO_2_ particles (T3 and T4) interacted with food or biological matrices.

Samples	Hydrodynamic Diameter (nm)							
	DDW	DDW			MEM	MEM		
		FBS	Albumin	Glucose		FBS	Albumin	Glucose
T3	304.7 ± 0.9 ^A,a^	404.8 ± 6.6 ^A,b^	397.5 ± 6.6 ^A,b^	317.9 ± 6.1 ^A,a^	440.9 ± 4.3 ^B,a^	505.6 ± 6.5 ^B,b^	495.3 ± 6.1 ^B,b^	456.3 ± 7.6 ^B,a^
T4	295.7 ± 2.9 ^A,a^	348.2 ± 1.4 ^A,b^	342.6 ± 14.8 ^A,b^	308.1 ± 5.9 ^A,a^	377.3 ± 2.8 ^B,a^	437.0 ± 9.2 ^B,b^	431.9 ± 2.6 ^B,b^	388.9 ± 1.0 ^B,a^

Different uppercase letters (A, B) next to values denote significant differences in hydrodynamic diameters interacting with FBS, albumin, or glucose in DDW compared to those in MEM (*p* < 0.05). Different lowercase letters (a, b) next to values denote significant differences among different matrices prepared in DDW or MEM (*p* < 0.05). Abbreviations: DDW, distilled deionized water; MEM, minimum essential medium; FBS, fetal bovine serum.

## Data Availability

The data presented in this study are available in the article.

## Supplementary Materials

The following supporting information can be downloaded at: https://www.mdpi.com/article/10.3390/ijms26020617/s1.
