# Supplementary material for: Effects of the Interactions Between Food Additive Titanium Dioxide and Matrices on Genotoxicity
Source: Int J Mol Sci. 2025 Jan 13;26(2):617. doi: 10.3390/ijms26020617 (PMC11765690; doi:10.3390/ijms26020617)
Supplement: Supplementary file 1 [file ijms-26-00617-s001.zip › ijms-3380671-supplementary.pdf]

International Journal of *Molecular Sciences*

MDPI

Supplementary materials

Effects of the interactions between food additive titanium dioxide and matrices on genotoxicity

Su-Min Jeong, Han-Na Nam and Soo-Jin Choi *

Department of Food Science & Technology, Seoul Women's University, Seoul 01797, Korea; zzzin_pang@swu.ac.kr (S.-M.J.); nha0318@swu.ac.kr (H.-N.N.)
* Correspondence: sjchoi@swu.ac.kr; Tel.: +82-2-970-5634; Fax: +82-2-970-5977

Table S1. Compositions of simulated digestion fluids of the in vitro digestion model [53].

Digestion fluids	pH	Composition<br>(amounts based on 0.4 L of digestion juices<br>at a 1.25× concentration)
Simulated saliva fluid (SSF)	7	0.5 M KCl (15.1 mL), 0.5 M KH_2PO_4 (3.7 mL), 1 M $NaHCO_3$ (6.8 mL), 0.15 M $MgCl_2(H_2O)_6$ (0.5 mL), 0.5 M $(NH_4)_2CO_3$ (0.06 mL), 6 M HCl (0.09 mL), 0.3 M $CaCl_2(H_2O)_2$ (0.025 mL)<br>Salivary amylase (75 U/mL)
Simulated gastric fluid (SGF)	3	0.5 M KCl (6.9 mL), 0.5 M KH_2PO_4 (0.9 mL), 1 M $NaHCO_3$ (12.5 mL), 2 M NaCl (11.8 mL), 0.15 M $MgCl_2(H_2O)_6$ (0.4 mL), 0.5 M $(NH_4)_2CO_3$ (0.5 mL), 6 M HCl (1.3 mL), 0.3 M $CaCl_2(H_2O)_2$ (0.005 mL)<br>Pepsin (2,000 U/mL), Gastric lipase (60 U/mL)
Simulated intestinal fluid (SIF)	7	0.5 M KCl (6.8 mL), 0.5 M KH_2PO_4 (0.8 mL), 1 M $NaHCO_3$ (42.5 mL), 2 M NaCl (9.6 mL), 0.15 M $MgCl_2(H_2O)_6$ (1.1 mL), 6 M HCl (0.7 mL), 0.3 M $CaCl_2(H_2O)_2$ (0.04 mL)<br>Trypsin in pancreatin (100 U/mL), Bile salts (10 mM)
